# Human Disturbance Influences Reproductive Success and Growth Rate in California Sea Lions (*Zalophus californianus)*

**DOI:** 10.1371/journal.pone.0017686

**Published:** 2011-03-16

**Authors:** Susannah S. French, Manuela González-Suárez, Julie K. Young, Susan Durham, Leah R. Gerber

**Affiliations:** 1 Department of Biology and the Ecology Center, Utah State University, Logan, Utah, United States of America; 2 Ecology, Evolution and Environmental Science, School of Life Sciences, Arizona State University, Tempe, Arizona, United States of America; 3 Laboratoire d'Ecologie et Evolution CNRS - UMR 7625, Université Pierre et Marie Curie, Paris, France; Texas A&M University, United States of America

## Abstract

The environment is currently undergoing changes at both global (e.g., climate change) and local (e.g., tourism, pollution, habitat modification) scales that have the capacity to affect the viability of animal and plant populations. Many of these changes, such as human disturbance, have an anthropogenic origin and therefore may be mitigated by management action. To do so requires an understanding of the impact of human activities and changing environmental conditions on population dynamics. We investigated the influence of human activity on important life history parameters (reproductive rate, and body condition, and growth rate of neonate pups) for California sea lions (*Zalophus californianus*) in the Gulf of California, Mexico. Increased human presence was associated with lower reproductive rates, which translated into reduced long-term population growth rates and suggested that human activities are a disturbance that could lead to population declines. We also observed higher body growth rates in pups with increased exposure to humans. Increased growth rates in pups may reflect a density dependent response to declining reproductive rates (e.g., decreased competition for resources). Our results highlight the potentially complex changes in life history parameters that may result from human disturbance, and their implication for population dynamics. We recommend careful monitoring of human activities in the Gulf of California and emphasize the importance of management strategies that explicitly consider the potential impact of human activities such as ecotourism on vertebrate populations.

## Introduction

Increasing rates of human population growth and anthropogenic impacts on a global scale have left few populations of plants and animals undisturbed. Participation in non-consumptive wildlife activities, such as eco-tourism, that generally do not directly harm organisms or their habitats is projected to double over the next 50 years [1]. Thus, human interactions with plants and animals may be among the most pressing issues in developing sustainable approaches to mitigating anthropogenic impacts. Yet most research that monitors populations at risk of decline or extinction has focused on behavioral and demographic measures of viability, without integrating human activity patterns. Moreover, understanding the mechanisms by which human activities affect reproduction and development may suggest novel approaches to mitigate the deleterious effects of these activities on wild populations.

Anthropogenic disturbance is a relevant and widespread facilitator of environmental change, with potentially significant implications for individuals and populations. There is increasing evidence that vertebrate populations are stressed when exposed to humans, which is manifested by changes in behavior and physiology. Williams et al. [2] found that human disturbance increased energetic costs as a result of behavioral modifications in killer whales (*Orcinus orca*). Similarly, energy expenditure significantly increased in brown bears (*Ursus arctos*) that were experimentally exposed to tourism [3]. Human disturbance also alters individual spatial distribution [4]–[5] and behavior [6] of animal populations. Behavioral and energetic changes are likely coupled with physiological alterations in the organism [7].

It has been widely demonstrated that human interactions with free-living vertebrates can lead to physiological stress (i.e., physiological response to a stressor, or stimulus). For example, marine iguanas exhibit changes in circulating concentrations of corticosterone in response to exposure to tourism [7]. Exposure to humans also impacts the stress physiology (i.e., hypothalamic-pituitary-adrenal axis activity and myriad of physiological changes that occur in response to stressor) of many other species such as Magellanic penguins (*Spheniscus magellanicus*;) [8]–[9], neotropical hoatzins (*Opisthocomus hoazin*) [10], wolves (*Canis lupus*) [11], and elk (*Cervus canadensis*) [11]. Behavior, energy availability, and physiological state are all important contributors to reproductive success and survival. Therefore perturbations to any of these components may affect individual fitness and ultimately lead to population declines [5].

**Figure 1:**
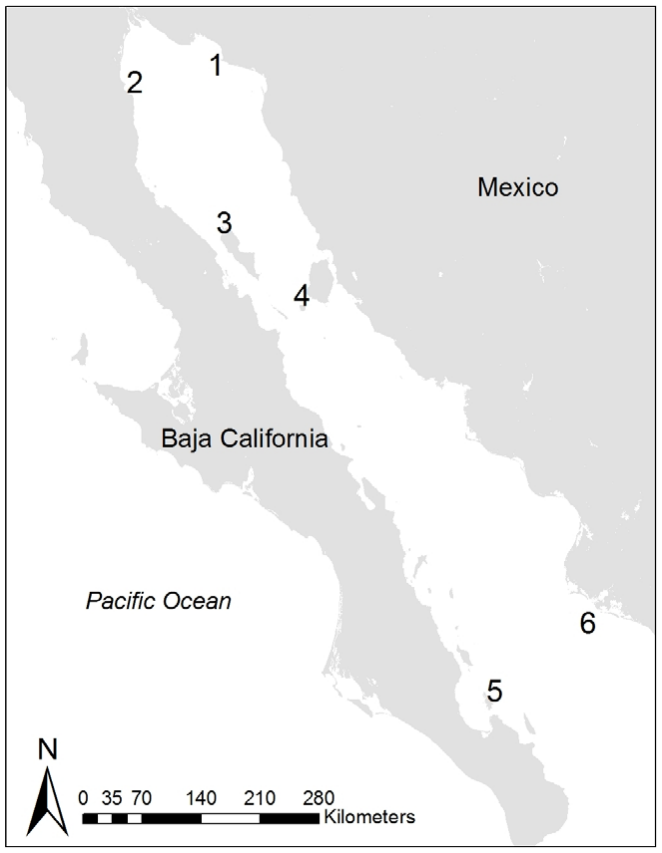
Map of the Gulf of California, Mexico. The studied California sea lion colonies are: (1) San Jorge, (2) Los Lobos, (3) Granito, (4) San Esteban, (5) Los Islotes, and (6) Farallon de San Ignacio.

An important step in developing effective conservation strategies for natural populations is to identify the impact of human activity on parameters critical to population sustainability, such as reproductive output. Reproduction is a relevant population parameter because it is relatively easy to measure in the field and is a vital component directly affecting population dynamics. In this study we explored how reproductive rate, pup body condition, and pup growth rate were affected by frequency of human activity in several breeding colonies of the California sea lion (*Zalophus californianus*) in Mexico (Figure 1).

California sea lions represent a useful model species to examine the effects of human exposure on wild populations for several reasons. First, sea lions typically aggregate in large groups during the reproductive season [12]. Consequently distinct populations occur within a fairly narrow geographical range and are appealing targets for ecotourism ventures that have thrived in locations such as the Gulf of California, Mexico [13]. Second, sea lion populations are exposed to increasing rates of human exposure that vary among colonies [13]–[14]. As many as 20 visitors per hour have been observed at a single breeding colony in this region the Gulf of California, with the number of visitors increasing considerably in recent years [13]. Sea lions are afforded some protection because islands in the Gulf of California currently are protected from human activity [15]–[16]; yet little enforcement occurs [17]. Although tourism is likely increasing on most islands in the Gulf of California, increases in human activities have occurred at different rates among islands. Thus, the distribution of study sites provides a natural experiment, where many environmental factors are similar but frequency of human exposure varies. Finally, our results could have implications for the conservation and management of this species. The total population of sea lions in the Gulf has declined by more than 20% in the last decade [18]. Understanding how frequency of human activity affects reproduction may provide insights into the causes of this decline and suggest measures to effectively conserve this population.

We first examine reproductive rates of California sea lions at 6 islands, each experiencing different degrees of human exposure over time (Figure 1). We also evaluate the relationship between frequency of human exposure and pup growth rate and body condition because the influence of disturbance may be manifested from reproduction into early development. Finally, we explore the potential effects of changes in human activities on the long-term population growth rate by developing a projection matrix based on our observed relationship between frequency of human exposure and reproductive rate. This matrix model was used to illustrate the relationship between fecundity (reproductive rate) and population health, which has been shown to vary depending on life history strategy [19]–[21]. Based on previous research, we predict that populations experiencing high levels of human exposure should exhibit comparably lower reproductive rates, and that neonates should have reduced body condition and decreased growth rates due to higher levels of stress both of the mothers (during pregnancy and after birth) and of the neonates themselves [22]–[24].

## Results

### Reproductive rate

**Figure 2:**
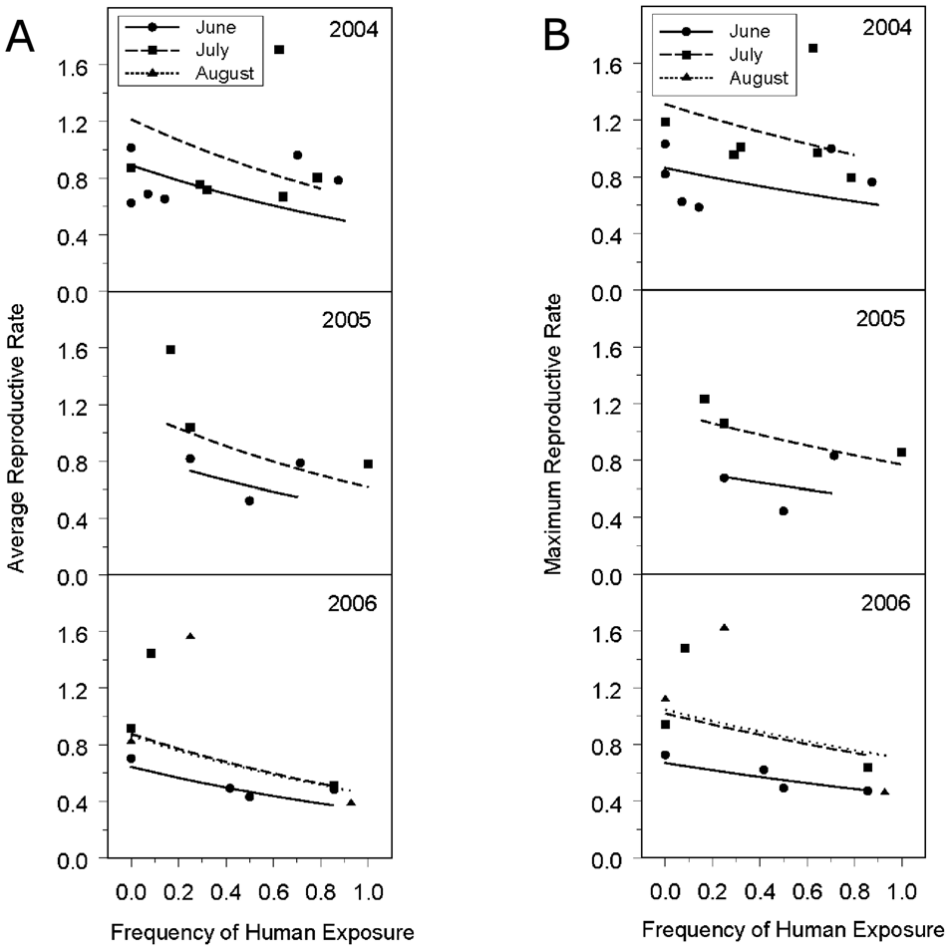
California sea lion reproductive rate versus frequency of human exposure. Reproductive rate and human exposure frequency during June, July and August in 3 years: (A) average reproductive rate; (B) maximum reproductive rate. Symbols depict observed data; the curve shows model predictions that are back-transformed to depict the regression of reproductive rate (pups/females) on frequency of human exposure (days with observed human presence/number of observation days in scanning period). Each curve represents a mean of the curves for the individual islands.

As predicted, both average and maximum reproductive rates declined with increasing human exposure (Figure 2). The estimated slope for the linear regression of reproductive rate (pups/females on ln scale) versus frequency exposure to humans (as a proportion) was 0.643 (SE = 0.236; *t*_4_ = 2.73; *P* = 0.053) for the average rate, and 0.399 (SE = 0.195; *t*_4_ = 2.04; *P* = 0.111) for the maximum rate (estimated coefficients and standard errors reported in Table S1). Average pup to female rates were higher in July and August compared to June (main effect of month *F*_2,13_ = 5.06, *P* = 0.024) and higher in 2004 and 2005 than in 2006 (main effect of year *F*_2,13_ = 3.59, *P* = 0.057). Maximum pup to female rates were also higher in July and August compared to June (main effect of month *F*_2,13_ = 9.46, *P* = 0.003). Although maximum reproductive rates were similar in pattern to average reproductive rates, being highest in 2004, intermediate in 2005, and lowest in 2006, maximum rates were not different among years (main effect of year *F*_2,13_ = 2.04, *P* = 0.170).

### Pup body condition

Male body condition (mean  =  0.00202 kg cm^−3^, SE = 0.00020 kg cm^−3^) was better than female body condition (mean =  0.00197 kg cm^−3^, SE = 0.00020) (*F*_1,31_ = 3.85, *P* = 0.059). There was no evidence of differences due to frequency of human exposure (*F*_1,3_ = 0.01, *P* = 0.940), year (*F*_2,31_ = 0.65, *P* = 0.528), or month (*F*_1,31_ = 0.58, *P* = 0.454; estimated coefficients and standard errors reported in Table S2).

### Pup growth rate

**Figure 3:**
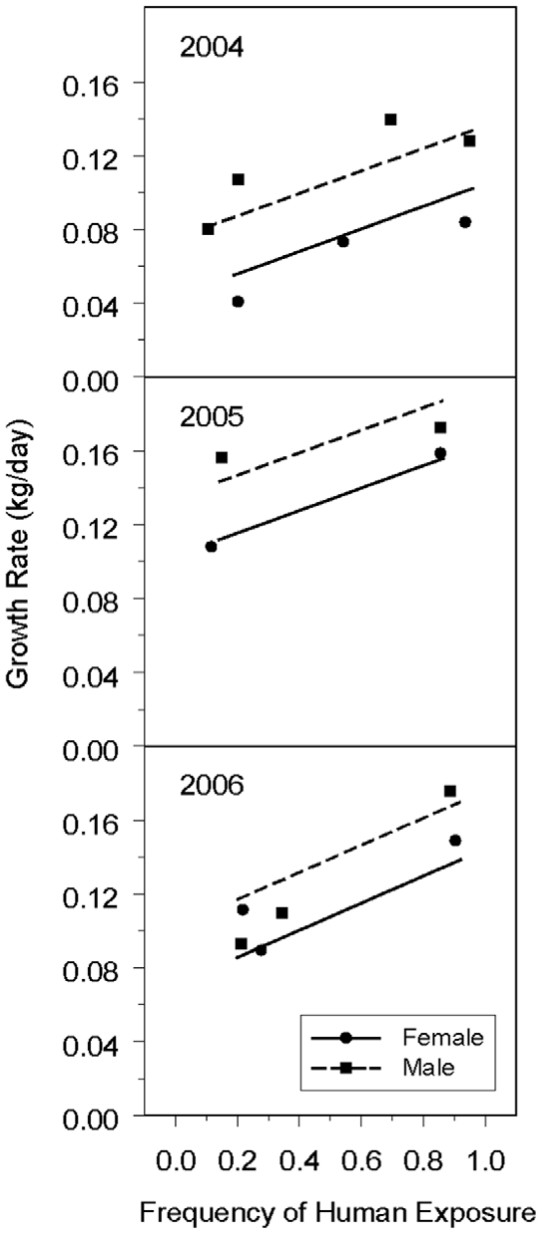
California sea lion pup growth rates versus frequency of human exposure. Growth rate and human exposure frequency for males (M) and females (F) in 3 years. Symbols depict observed data; the curve depicts the regression of growth rate (kg/day) on frequency of human exposure (days with observed human presence/number of observation days in scanning period).

Contrary to our expectations, pup growth rate increased with increasing exposure to humans (Figure 3). The estimated slope for the linear regression of growth rate (kg/day) versus frequency of exposure to humans (as a proportion) was 0.0735 (SE = 0.0144; *t*_2_ = 5.11; *P* = 0.032; estimated coefficients and standard errors reported in Table S3). Growth rates were higher for males (mean  =  0.136 kg day^−1^, SE = 0.0059) than for females (mean =  0.105 kg day^−1^, SE = 0.0061) (*F*_1,7_ = 13.99, *P* = 0.007). Growth rates were lowest in 2004, highest in 2005, and intermediate in 2006 (*F*_2,7_ = 16.05, *P* = 0.002), although 2005 and 2006 were not shown to be different. Each line in Figure 3 represents a mean over the regression lines for the individual islands. Variability in individual observations that may appear excessive in this figure is addressed by the model and does not necessarily indicate lack of fit.

### Long-term annual population growth rate (λ)

**Figure 4:**
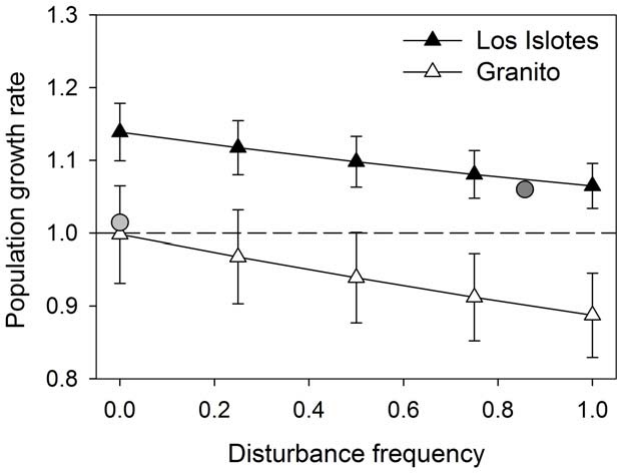
Predicted changes in annual population growth rates (λ) due to varying frequencies of human exposure. Triangles represent estimated λs for mean survival and growth rates. Error bars are λ's estimated for the upper and lower 95% confidence interval values of the survival and growth rates. Grey circles indicate values of λ predicted for the observed reproductive rate and the observed frequency of human exposure in July 2006 (light grey: Granito; dark grey: Los Islotes). The dashed horizontal line separates the region of increasing populations (λ>1) from that of decreasing populations (λ<1).

To estimate reproductive rates we used island-specific regressions for July 2006, because 2006 was the last year of data collection and July is generally the best time to estimate reproductive rates as most females spend long periods in the colonies and all pups have been born. Using regression coefficients from other months or years did not qualitatively change the results. Based on the random coefficient model analysis the average reproductive rate at Los Islotes was exp(0.020–0.643*frequency human exposure) and at Granito was exp(−0.169-0.643*frequency human exposure). These regressions were used to predict reproductive rates under a range of human exposure frequencies (see Methods). All predicted reproductive rates based on these regressions and the range of human exposure frequencies considered were within the range of values observed in our study sites. For all estimates of survival and growth rates and both colonies, increasing the frequency of human exposure resulted in a large decrease in predicted long-term annual population growth rates (Figure 4). However, the population at Los Islotes, where frequency of human exposure is currently high (see observed value in Figure 4), maintained an increasing population growth trend (λ>1) even at the highest level of human exposure. In contrast, at Granito, where observed human exposure is currently low (see observed value in Figure 4), increases in the frequency of human exposure were predicted to quickly lead to a declining population (λ<1).

## Discussion

### Reproductive rate

We examined frequency of human exposure and its relationship to reproductive rates and estimates of pup growth and condition in several colonies of the California sea lion in the Gulf of California, Mexico. Our results suggest that reproductive rates are reduced by increases in the frequency of human exposure estimated as the proportion of days in which human presence was observed in several sea lion colonies. This effect was significant only for the mean rate, but the trend was also negative for the maximum rate, providing further support to the existence of a link between human presence and reduced reproduction. These results indicate that exposure to humans can be clearly defined as a disturbance for California sea lions. In addition, we found that both measures of reproductive rate vary according to month, which is explained by the highly seasonal reproductive process of sea lions. In fact, the peak in pup production occurs mid- to end of June [12], [25].

A potential mechanism for decreased reproduction is the effect of stress in response to human presence [26]–[28]. Decreased reproductive success caused by human disturbance has been observed in other species, including other pinnipeds [29]–[34]. Although our study did not specifically address the physiological mechanisms, stressful exposure to humans may alter specific hormone concentrations [7]–[9], which in turn affect physiological processes and reproduction. Future studies should examine baseline and stress-induced hormone levels (e.g., glucocorticoids) to identify the mechanisms altering reproduction in populations of California sea lions in the Gulf of California.

### Pup condition and growth

Pup body condition, however, was apparently not influenced by frequency of human exposure. In our study, body condition was determined for very young pups and primarily reflected prenatal growth or condition at birth. Previous studies have shown that birth weights in California sea lions are not directly related to maternal size [35] or to environmental conditions such as El Niño events [36]. Therefore, it is possible that the weight at birth is not a flexible trait which responds to external conditions. Instead it may be tightly controlled such that pups are born in optimum condition or may be restricted due to developmental constraints. Other studies also have found no effect of human disturbance on early postnatal weights in species such as the yellow-eyed penguin (*Megadyptes antipodes*) [37] or the eastern bluebird (*Sialia sialis*) [38].

Consistent with previous research, our results indicate that both pup growth rate and body condition are gender-specific, with males growing at a faster rate and having better body condition than females [35], [39]. However, contrary to what we anticipated, increases in the frequency of human exposure were associated with greater pup growth rates (Figure 3). Yearly differences in resources can greatly influence the growth potential of individuals [36]. The observed reduction in reproductive rates could increase the resources available for newborns, resulting in increased growth rates. Frequency of human exposure may indirectly influence pup development by modifying population densities which, via competition for resources, affect food resource availability (e.g., either indirectly via mother nursing capability or directly via pup food consumption) which is important for pup growth and development.

Alternatively, hormones important to the growth and development of individuals are known to be stress sensitive (e.g., growth hormone, somatostatin) [40], [41]. Therefore, if frequency of human exposure is indeed changing stress physiology in sea lions, it could contribute to altered offspring growth rates. More research is needed to measure circulating adrenal steroids, key mediators of physiological stress, and related hormones important for growth and development (i.e., growth hormone). Lastly, human exposure may be selecting for the type of adult sea lions that remains at highly disturbed sites, whereby the type of sea lions that survive and reproduce retain the traits we observed. For example, at disturbed sites fewer individuals are able to reproduce, but those that are reproductively active may produce more viable and faster growing offspring. Future research is necessary to clarify the relationship between pup development and human disturbance and to investigate the potential mechanisms regulating this relationship.

In general, our results suggest that exposure to humans may influence offspring development and reproductive output in California sea lion populations. Although the effects of human disturbance on reproduction and pup growth appear contradictory, the mechanisms may be related. For example, chronic stress in response to human presence may decrease reproductive rates [27], [42] if stressed females are less able to get pregnant or carry offspring to term. Offspring growth, however, likely is more dependent on available resources [12], [35], [36], [43], [44]. With fewer offspring being produced, resources may be more readily available for nursing mothers, which in turn may result in increased growth rates for pups. While some of our results (i.e., pup growth rates) suggest that increased disturbance may have a positive effect on the population, the negative impact of disturbance on reproductive rate could counterbalance this benefit and reduce population viability. How these factors interact to determine the long-term viability of the population remains to be understood. Similarly, the potential long-term effects of disturbance in these populations are yet unknown.

While ecotourism or fishing activities that are critical to local economies may impact California sea lion populations, our population model results suggest that a reduced reproductive rate can lead to declining population trends. However, it is also possible that increasing pup growth rates could offset some of these costs. Previous studies in avian, mammalian, reptilian, and plant species show that the relationship between fecundity (reproductive rate) and population growth is not always apparent, and other measures such as survivorship may be more important in explaining population dynamics [19]–[21]. Nevertheless, we found that changes in reproductive rates linked to increases in human exposure frequency have the potential to decrease population growth rates by as much as 11% (Figure 4). It is also important to note that our goal was not to accurately predict population viability but rather to illustrate how changes in reproductive rates could translate into changes at the population level. Our model greatly simplified sea lion life-history, and more importantly we did not include environmental or demographic variation which is likely to influence population viability. Therefore, our results should not be interpreted as realistic predictions of future population growth, but rather an indication of the potential population level effects of changes in the frequency of human exposure.

In conclusion, our analyses suggest that increasing human exposure is a disturbance and that the reduction of human presence could be a potential management option to recover or protect sea lion colonies Humans are already influencing life-history traits that have the potential to significantly influence population dynamics. First, at a time when human pressure is increasing in the Gulf of California [13]–[14], we highlight the importance of monitoring human activities carefully and to continue to assess their effects on sea lion populations. Second, future research should target the mechanisms by which human presence is affecting reproduction and pup growth rates, to ensure adequate protection and promote sustainable human-sea lion interactions. Finally, we recommend the introduction, implementation, and enforcement of management policies designed to protect sea lion populations from the negative impacts of human presence.

## Methods

### Ethics Statement

This study was carried out in strict accordance with the recommendations in the Guide for the Care and Use of Laboratory Animals of the National Institutes of Health. All animal procedures were approved by the Arizona State University Institutional Animal Care and Use Committee (protocol 07-918R).

### Data collection

#### Study sites

We collected data at 2 breeding sites on each of 6 different islands distributed throughout the Gulf of California (Figure 1) in 2004–2006. Our sampling represents nearly 50% of all islands identified as breeding colonies for this species in the region (n = 13) [18]. These islands were selected to cover the geographical range of the Gulf of California, from San Jorge in the north (31° 01′ N, 113° 15′ W) to Los Islotes in the south (24° 35′N, 110° 23′ W) and to represent varying degrees of exposure to humans largely based on proximity to developed areas along the Baja peninsula and mainland of Mexico. In addition, we chose islands with similar terrain and adequate accessibility.

#### Demographic data

Abundance data were collected at each site 4 to 6 times per day (from 0700 hr to 1900 hr) in scanning periods of 2 to 8 days during the summers of 2004–2006 (Table 1). Observers counted the total number of sea lions in the following age groups: adult males, adult females, sub-adult males, juveniles, and pups. These demographic categories were identified based on definitions established by LeBouef et al. [45]. All observers were trained to identify sea lion age groups accurately prior to data collection. More details of the study sites and general methodology can be found in [46]–[49].

**Table 1:** Time table of data collection on California sea lions across islands Baja, Mexico.

Island	Jun-04	Jul-04	Jun-05	Jul-05	Jun-06	Jul-06	Aug-06^a^
San Jorge	x	x	x	x	x	x	x
Isla Lobos	x	x					
Granito	x	x	x^b^	x	x	x	x
San Esteban	x	x			x^a^^,^ b		
Farallón de San Ignacio	x^2^	x					
Los Islotes	x	x	x	x	x	x	x

a No pup body condition data.

b No growth rate data.

Using the count data we computed 2 measures of reproductive rate for each scanning period at each site: 1) the ratio of the maximum number of pups to the maximum number of females, and 2) the ratio of the average number of pups to the average number of females (over observation periods within days and scanning periods). Although the 2 measures were strongly correlated (see results below), there are practical arguments for including both measurements of reproductive rate. Previous research shows that the number of pups is frequently underestimated in population counts [45], and therefore maximum rate may be more accurate than average rate. Specifically, pups often rest amongst or under large rocks and boulders which makes it difficult to count the entire pup population from fixed locations. Similarly, the total number of females is likely to be underestimated because individuals foraging at sea are not counted. On the other hand, the average is a more intuitive measure and captures data from all counts whereas the maximum is a single estimate.

Any proportional bias in counts of both pups and females likely was similar across sites because sites were chosen to comprise similar habitat features (rocky shores) [50]; observation locations were chosen to maximize visibility; and all observers used the same survey method. A previous study also suggests that bias in pup and adult counts is comparable across sites in the Gulf of California [47].

#### Human Exposure Measures

We noted the presence or absence of human activities at a site immediately prior to each demographic count. Human presence (i.e., exposure) was defined as the observation of any boats or humans (divers, swimmers) within 50 m of the coastline of the observation sites. A distance of 50 m was used because previous research [51] and our personal observations indicate that human presence at greater distances does not generate disturbance in sea lion colonies. An overall human exposure frequency for each scanning period and site was calculated based on the rate of days in which human presence was observed at least once divided by the number of observation days in the scanning period.

#### Pup Data

We captured and measured between 25–80 pups at each site during ∼3 days after each scanning period. Pups were weighed to the nearest 0.5 kg using a Pesola scale, and total body length and thoracic girth were measured to the nearest cm to calculate condition and growth rates. After measuring, we shaved a unique code into the backs of neonate pups or applied uniquely numbered plastic tags (Rototag, Dalton Inc) to the front flippers of older pups (Rototag, Dalton Inc) for future identification. Typically, all pups captured in June received haircuts while pups captured in July were large enough to be tagged. Haircut codes were visible for at least 4 months and were replaced with plastic tags that lasted >1 year if the animals were recaptured. Individual identification allowed us to estimate growth rates for recaptured individuals. Although the capture protocol was developed to minimize disturbance to the colony, our activities disrupted the sea lion colonies temporarily. We cannot directly estimate the effect of our captures as data could not be obtained without handling the animals, and thus control comparisons are not possible. However, captures were conducted at the end of each field trip (i.e., after demographic and human exposure data were collected for multiple days) and all study sites were subject to the same capture activities; thus, our activities should not bias our results.

Links between energy reserves, body condition, growth, investment into reproduction, and health maintenance are well-established [52]–[56]. As an indicator of individual health, we used a standard estimate of body condition (BCI) for pinnipeds [57]–[59]: BCI =  m/V, where m  =  mass (in g), V  =  volume (in cm^3^) estimated as V =  0.0265⋅L⋅GT^2^, L =  length (in cm) and GT  = thoracic girth (in cm). Higher values of BCI correspond to better body condition. BCI was computed once for each pup based on measurements made at its initial capture in June or July. Most individuals are born during the month of June [25]; individuals were captured shortly after birth, and therefore the estimated BCI primarily reflected early postnatal condition. Although there is some variability in timing of the reproductive peak among islands, previous work demonstrated that births occur over an extended period without a strongly marked peak. Thus there is high overlap among colonies [25], and it is unlikely that potential differences in the timing of births among islands affected our results.

We also estimated postnatal growth rate based on the change in weight of individual pups that were recaptured 3–6 weeks after their initial captures. Growth rate (G, in kg/day) was calculated as G = (m_2_–m_1_)/T, where m_2_ = weight at recapture (in kg), m_1_ = weight at initial capture (in kg), and T = number of days between initial capture and recapture.

### Statistical analysis

As in most field studies, observational units (i.e., islands) in this study were not randomly selected. Although we do not consider islands to be a random sample, they are used in the statistical analysis *as if* they comprise a random sample, i.e., as replicates, and we interpreted our results accordingly. Consequently, we computed summary values for variables for each scanning period at each island, and used those values as data for analysis. Frequencies of human exposure were averaged over sites within an island. Reproductive rates were averaged over sites within each island and then log-transformed prior to analysis to better meet model assumptions. Mean pup growth rate and mean body condition were computed over all pups at each site, and those means were averaged for each island.

To assess the effect of human exposure on California sea lion population parameters, we linearly regressed each response variable (i.e., log-transformed average and maximum reproductive rates, pup growth rate, and pup body condition) on frequency of human exposure using a random coefficient model [59]–[60]. The random coefficient model fitted a regression for each island using measurements through time as data; the regression lines for the islands comprised a random sample from which an estimate of the true population regression line was derived. Year (for reproductive rates, pup growth rate, and pup body condition), month (for reproductive rates and pup body condition), and gender (for pup growth rate and pup body condition) were included as main effects in the model to assess the effects of these explanatory factors on the intercepts of the regressions. Complete data collections were not obtained in all cases (Table 1), the structure of the available data placed limits on the number of parameter estimates. Therefore no interactions among explanatory factors were included in the model, and we did not attempt to model the temporal covariance among the repeated measures on each island. Assessments of pair wise comparisons among month and year means were based on adjusted *P*-values obtained using the Tukey-Kramer method with family-wise Type I error rate (FWER) set at 0.10, given that the Tukey-Kramer test is known to be conservative [61]. Determination of significance of statistical tests followed the “neoFisherian significance assessment” approach espoused by [62]. Based on residual analysis, we determined that the assumptions of normality, homogeneity of variance, and linearity were adequately met for all models; no outliers were identified. Analyses were generated using the MIXED procedure in SAS/STAT® software, Version 9.1.3 of the SAS System for Windows.

We could not assess individual-level responses of pups to human activity due to a mismatch in measurement scale: frequency of human exposure was measured at the site level whereas pup growth and body condition were measured at the individual level.

### Population growth model

To examine the effects of individual life-history on overall population dynamics, we investigated whether the predicted variation in reproductive rate associated with changes in the frequency of human exposure affected the estimated long-term annual population growth rate (λ). We followed Caswell [63] to estimate λ as the dominant eigenvalue of a projection matrix assuming a constant environment for simplicity. The projection matrix was defined as a simple stage-structure model with 3 stages describing California sea lion life history: pups (0–1 year-old), juveniles (1–4 year-old), and adults (>4 year-old). These stages correspond to the demographic categories used during field observations. The model assumed a 1-year transition and required estimates of adult reproductive rates (only adults breed), survival rates (probability of remaining in the same stage) for juveniles and adults, and growth rates (probability of moving to the next life stage) for all 3 stages. Wielgus et al. [64] applied a data-fitting technique that used stage-specific abundance data to estimate demographic rates for these 3 stages. Based on their estimates we defined 3 basic projection matrices per island using the mean, upper, and lower 95% confidence interval values of the survival and growth rates. Wielgus et al. [64] only estimates survival and growth rates for 2 of our study colonies: Los Islotes and Granito; thus, we only explored the effect of changes in the frequency of human exposure at these 2 locations. We used our resulting regression functions (see Results) to predict reproduction rates under 5 equally spaced human exposure frequencies: 0, 0.25, 0.5, 0.75, and 1. The 3 basic projection matrices per island were then combined with each of the 5 reproductive rate estimates to obtain 15 λ estimates for island. It is important to note that our λ predictions are not intended to accurately reflect future population growth but rather provide an insight into the potential population level effects of increased or decreased exposure to humans. First, survival and growth were inversely estimated from changes in population size and may be biased by movement between colonies [65]. Second, our estimates of reproductive rates were derived from a linear relationship and extrapolated to frequencies of human exposure beyond the observed values, therefore there is uncertainty in our predictions [66]. Finally, we assume a constant environment for simplicity but true assessments of population growth must incorporate environmental (and demographic) stochasticity. Nevertheless, our simplified model provides insights into the potential population level effects of changes in human exposure frequency.

## Supporting Information

Table S1 Estimated coefficients and standard errors by year and sex for linear regression of ln-transformed reproductive rate (pups/females) on frequency of human exposure (days with observed human presence/number of observation days in scanning period). The regression of reproductive rate on frequency human exposure is Reproductive rate  =  exp(Intercept + Slope × frequency human exposure).
(DOC)

Table S2 Estimated coefficients and standard errors by year and sex for linear regression of pup body condition (g cm^−3^) on frequency of human exposure (days with observed human presence/number of observation days in scanning period).
(DOCX)

Table S3 Estimated coefficients and standard errors by year and sex for linear regression of pup growth rate (kg/day) on frequency of human exposure (days with observed human presence/number of observation days in scanning period).
(DOCX)
