# Supplementary material for: Human Disturbance Influences Reproductive Success and Growth Rate in California Sea Lions (Zalophus californianus)
Source: PLoS One. 2011 Mar 16;6(3):e17686. doi: 10.1371/journal.pone.0017686 (PMC3059216; doi:10.1371/journal.pone.0017686)
Supplement: Table S1 — Estimated coefficients and standard errors by year and sex for linear regression of ln-transformed reproductive rate (pups/females) on frequency of human exposure (days with observed human presence/number of observation days in scanning period). The regression of reproductive rate on frequency human exposure is Reproductive rate = exp(Intercept + Slope × frequency human exposure). (DOC) [file pone.0017686.s001.doc]

| Year | Month | Intercept | (SE) | Slope | (SE) |
| --- | --- | --- | --- | --- | --- |
| Average reproductive rate | |  |  |  |  |
| 2004 | June | -0.1143 | (0.1644) | -0.6430 | (0.2357) |
| 2004 | July | 0.1954 | (0.1657) | -0.6430 | (0.2357) |
| 2005 | June | -0.1502 | (0.1844) | -0.6430 | (0.2357) |
| 2005 | July | 0.1595 | (0.1874) | -0.6430 | (0.2357) |
| 2006 | June | -0.4396 | (0.1711) | -0.6430 | (0.2357) |
| 2006 | July | -0.1299 | (0.1771) | -0.6430 | (0.2357) |
| 2006 | August | -0.1465 | (0.1990) | -0.6430 | (0.2357) |
| Maximum reproductive rate | |  |  |  |  |
| 2004 | June | -0.1476 | (0.1273) | -0.3989 | (0.1954) |
| 2004 | July | 0.2720 | (0.1293) | -0.3989 | (0.1954) |
| 2005 | June | -0.2814 | (0.1552) | -0.3989 | (0.1954) |
| 2005 | July | 0.1381 | (0.1584) | -0.3989 | (0.1954) |
| 2006 | June | -0.4009 | (0.1397) | -0.3989 | (0.1954) |
| 2006 | July | 0.0186 | (0.1473) | -0.3989 | (0.1954) |
| 2006 | August | 0.0443 | (0.1743) | -0.3989 | (0.1954) |
