# Supplementary material for: Human Disturbance Influences Reproductive Success and Growth Rate in California Sea Lions (Zalophus californianus)
Source: PLoS One. 2011 Mar 16;6(3):e17686. doi: 10.1371/journal.pone.0017686 (PMC3059216; doi:10.1371/journal.pone.0017686)
Supplement: Table S2 — Estimated coefficients and standard errors by year and sex for linear regression of pup body condition (g cm−3) on frequency of human exposure (days with observed human presence/number of observation days in scanning period). (DOCX) [file pone.0017686.s002.docx]

| Year | Month | Sex^a^ | Intercept | (SE) | Slope | (SE) |
| --- | --- | --- | --- | --- | --- | --- |
| 2004 | June | F | 1.9501 | (0.03258) | 0.003368 | (0.04122) |
| 2004 | June | M | 2.0041 | (0.03268) | 0.003368 | (0.04122) |
| 2004 | July | F | 1.9710 | (0.03246) | 0.003368 | (0.04122) |
| 2004 | July | M | 2.0250 | (0.03257) | 0.003368 | (0.04122) |
| 2005 | June | F | 1.9812 | (0.03782) | 0.003368 | (0.04122) |
| 2005 | June | M | 2.0352 | (0.03793) | 0.003368 | (0.04122) |
| 2005 | July | F | 2.0021 | (0.03771) | 0.003368 | (0.04122) |
| 2005 | July | M | 2.0561 | (0.03781) | 0.003368 | (0.04122) |
| 2006 | June | F | 1.9413 | (0.03773) | 0.003368 | (0.04122) |
| 2006 | June | M | 1.9953 | (0.03783) | 0.003368 | (0.04122) |
| 2006 | July | F | 1.9621 | (0.03761) | 0.003368 | (0.04122) |
| 2006 | July | M | 2.0162 | (0.03772) | 0.003368 | (0.04122) |

^a^F=female, M=male
