# Supplementary material for: Human Disturbance Influences Reproductive Success and Growth Rate in California Sea Lions (Zalophus californianus)
Source: PLoS One. 2011 Mar 16;6(3):e17686. doi: 10.1371/journal.pone.0017686 (PMC3059216; doi:10.1371/journal.pone.0017686)
Supplement: Table S3 — Estimated coefficients and standard errors by year and sex for linear regression of pup growth rate (kg/day) on frequency of human exposure (days with observed human presence/number of observation days in scanning period). (DOCX) [file pone.0017686.s003.docx]

| Year | Sex | Intercept | (SE) | Slope | (SE) |
| --- | --- | --- | --- | --- | --- |
| 2004 | Female | 0.0375 | (0.01103) | 0.0735 | (0.01438) |
| 2004 | Male | 0.0689 | (0.01040) | 0.0735 | (0.01438) |
| 2005 | Female | 0.0971 | (0.01198) | 0.0735 | (0.01438) |
| 2005 | Male | 0.1284 | (0.01184) | 0.0735 | (0.01438) |
| 2006 | Female | 0.0709 | (0.01072) | 0.0735 | (0.01438) |
| 2006 | Male | 0.1023 | (0.01056) | 0.0735 | (0.01438) |
